# Metabolic Alterations in Cardiopulmonary Vascular Dysfunction

**DOI:** 10.3389/fmolb.2018.00120

**Published:** 2019-01-22

**Authors:** Valérie Françoise Smolders, Erika Zodda, Paul H. A. Quax, Marina Carini, Joan Albert Barberà, Timothy M. Thomson, Olga Tura-Ceide, Marta Cascante

**Affiliations:** ^1^Department of Biochemistry and Molecular Biology and Institute of Biomedicine (IBUB), Faculty of Biology, University of Barcelona, Barcelona, Spain; ^2^Department of Pulmonary Medicine, Hospital Clínic-Institut d'Investigacions Biomèdiques August Pi I Sunyer (IDIBAPS), University of Barcelona, Barcelona, Spain; ^3^Einthoven Laboratory for Experimental Vascular Medicine, Leiden University Medical Center, Leiden, Netherlands; ^4^Department of Vascular Surgery, Leiden University Medical Center, Leiden, Netherlands; ^5^Department of Pharmaceutical Sciences, Università degli Studi di Milano, Milan, Italy; ^6^Centro de Investigación Biomédica en Red (CIBER) de Enfermedades Respiratorias, Madrid, Spain; ^7^Institute for Molecular Biology of Barcelona, National Research Council (IBMB-CSIC), Barcelona, Spain; ^8^Centro de Investigación Biomédica en Red (CIBER) de Enfermedades Hepáticas y Digestivas, Madrid, Spain

**Keywords:** acute myocardial infarction, pulmonary hypertension, endothelial dysfunction, cellular metabolism, glycolysis, metabolic targets, systems biology

## Abstract

Cardiovascular diseases (CVD) are the leading cause of death worldwide. CVD comprise a range of diseases affecting the functionality of the heart and blood vessels, including acute myocardial infarction (AMI) and pulmonary hypertension (PH). Despite their different causative mechanisms, both AMI and PH involve narrowed or blocked blood vessels, hypoxia, and tissue infarction. The endothelium plays a pivotal role in the development of CVD. Disruption of the normal homeostasis of endothelia, alterations in the blood vessel structure, and abnormal functionality are essential factors in the onset and progression of both AMI and PH. An emerging theory proposes that pathological blood vessel responses and endothelial dysfunction develop as a result of an abnormal endothelial metabolism. It has been suggested that, in CVD, endothelial cell metabolism switches to higher glycolysis, rather than oxidative phosphorylation, as the main source of ATP, a process designated as the Warburg effect. The evidence of these alterations suggests that understanding endothelial metabolism and mitochondrial function may be central to unveiling fundamental mechanisms underlying cardiovascular pathogenesis and to identifying novel critical metabolic biomarkers and therapeutic targets. Here, we review the role of the endothelium in the regulation of vascular homeostasis and we detail key aspects of endothelial cell metabolism. We also describe recent findings concerning metabolic endothelial cell alterations in acute myocardial infarction and pulmonary hypertension, their relationship with disease pathogenesis and we discuss the future potential of pharmacological modulation of cellular metabolism in the treatment of cardiopulmonary vascular dysfunction. Although targeting endothelial cell metabolism is still in its infancy, it is a promising strategy to restore normal endothelial functions and thus forestall or revert the development of CVD in personalized multi-hit interventions at the metabolic level.

## Importance of Cardiopulmonary Diseases

Cardiovascular diseases (CVD) are leading cause of death in the world. In 2015, The World Health Organization (WHO) estimated that 17.7 million people died from CVD, representing 31% of all global deaths (Townsend et al., 2015). Death from CVD is associated with increasing age, with 1.4 million deaths in individuals under 75 and 700,000 in individuals under 65 (Townsend et al., 2016).

CVD encompass a range of diseases affecting the functions of the heart and blood vessels, driven by diverse underlying mechanisms. In this review, we will focus on acute myocardial infarction (AMI), a type of coronary artery disease, and pulmonary arterial (PAH), and chronic thromboembolic pulmonary hypertension (CTEPH). Both types of CVD share a diseased endothelium conducive to atherosclerosis and endothelial hyperproliferation, respectively, with consequent narrowing of arteries, compromised blood flow and reduced oxygen and nutrient supply to the vascular cells, eventually leading to cardiac hypertrophy and myocardial infarction.

### Atherosclerosis

Atherosclerosis, or the formation of atherosclerotic plaques, underlies one of the major causes of morbidity and mortality in developed and developing nations (Townsend et al., 2015). The World Health Organization attributes an estimated 16.7 million deaths to atherosclerotic cardiovascular disease (Leopold and Loscalzo, 2005; Hyder et al., 2007). Atherosclerotic plaque formation and rupture is one of most common pathogenic mechanisms of coronary artery disease, stroke, and peripheral artery disease (Grootaert et al., 2015). Atherogenesis is the result of complex sequences of events associated with processes such as endothelial dysfunction, neovascularization, vascular proliferation, apoptosis, matrix degradation, inflammation, oxidative stress, and thrombosis (Hansson, 2005). All these processes affect the inner lining of the artery. Eventually, arteries become thicker by virtue of an accumulation of calcium, fat deposits and inflammatory cells, leading to the formation of the atherosclerotic plaque (Liu et al., 2015). Although the pathophysiological mechanisms of atherosclerosis are yet to be fully unveiled, increasing evidences point to a critical role played by endothelial and metabolic dysregulation involving downregulation of oxidative phosphorylation and fatty acid metabolism. A complete understanding of endothelial metabolic reprogramming underlying atherosclerosis requires further investigation and could open new avenues in the prevention and treatment of this disease (Moreno-Viedma et al., 2016).

#### Current Therapies in Atherosclerosis and AMI

The four major risk factors for developing atherosclerosis are hypercholesterolemia, diabetes, hypertension, and cigarette smoking (Bergheanu et al., 2017). Recent studies have shown that an adequate control of lipoprotein levels reduces the risk of atherosclerosis events. As such, a modification in daily diet, an increase in physical activity and cessation of smoking constitute the cornerstones of any intervention aimed at the prevention and/or treatment of atherosclerosis. While the role of the other parameters is still not clear, TG (triglycerides), LDL-C (low density lipoprotein cholesterol), and HDL-C (high density lipoprotein cholesterol) remain very strong predictors of premature atherosclerosis (Catapano et al., 2016). Hypercholesterolemia alters vascular permeability, allowing the leaking of LDL cholesterol and deposition on the arterial walls. There, LDL is subject to modifications that include oxidation, enzymatic processing, and aggregation, that render the lipoprotein particles proinflammatory and induce an immune response. As part of this response, monocytes are recruited to the sub-endothelial space where they differentiate into macrophages. Macrophages may also derive from pluripotent cells associated with blood vessels. Regardless of their origin, macrophages in atherosclerotic lesions actively participate in lipoprotein ingestion and accumulation giving rise to foam cells filled with cholesterol-rich lipid droplets. These processes lead to vascular modifications visible as fatty streaks, intimal thickening and ruptured plaques, causing acute coronary disease (Bergheanu et al., 2017). Vulnerable plaques contain monocytes, macrophages and T cells, which accounts for their instability.

The critical role played by LDL-C in atherosclerosis has prompted the development of rational strategies to counter the pathogenic effects of hypercholesterolemia. The use of statins (inhibitors of HMG-CoA reductase, the rate-controlling enzyme of the mevalonate, and cholesterol synthesis pathways) as a pharmacological approach to lower cholesterol levels is one of the most widely used therapies in the treatment of atherosclerosis and acute coronary syndromes, as these drugs show consistent clinical event reductions with a very good safety profile (Adams et al., 2014). Other clinical studies reveal that the use of ezetimibe (an inhibitor of intestinal cholesterol absorption) considerably reduces LDL-C blood levels when combined with statins (Catapano et al., 2016). An alternative approach is the administration of fibrates, a particular class of agonists of the peroxisome proliferator-activated receptor α (PPAR-α) a regulator of lipoprotein metabolism, showing a good effect in lowering TG levels as mono-therapy, even though the results are not as promising as for the statins (Adams et al., 2014). Recently, a large multi-scale and multiethnic study was undertaken to better understand the role of genetic and environmental factors in CVD and to identify genetic variants associated to blood lipids levels (Klarin et al., 2018). By using a genome wide association study (GWAS) on Million Veterans Program (MVP), this work depicted some novel lipid associated coding variants with CVD risk or incidence. Individuals with mutations in ANGPTL4 (Angiopoietin-like4) presented a lower risk to develop diabetes mellitus (type2), those with a loss of function in PCSK9 (Proprotein convertase subtilisin/kexin type 9) showed a reduced risk of abdominal aortic aneurysm, and those treated with inhibitors of PDE3B (Phosphodiesterase 3B), presented reduced levels of triglycerides in blood (Cohen et al., 2006; Willer et al., 2013; Ahmad et al., 2016; Dewey et al., 2017; Graham et al., 2017). These data highlight the complexity in humans to control blood lipid composition and the potential of a genetic approach to develop novel therapeutic agents for the prevention of cardiovascular disease.

When the combination of healthier diet, lifestyle and pharmacological treatments fail to improve the pathological and clinical conditions in atherosclerotic patients with an associated coronary disease, surgical intervention is considered the best option. Coronary artery thrombosis with complete occlusion typically leads to ST-segment elevation myocardial infarction (STEMI). Partial occlusion, or occlusion in the presence of collateral circulation, results in non-STEMI or unstable angina, an acute coronary syndrome without ST-segment elevation. Once a definitive or likely diagnosis of an acute coronary syndrome without ST-segment elevation has been made, the patient is triaged to either an invasive strategy or an ischemia-guided strategy. An invasive strategy leads to improved outcomes and is favored for the majority of patients (Anderson and Morrow, 2017).

In patients presenting with an unstable condition or with STEMI, urgent Percutaneous Coronary Intervention (PCI) is performed. An ischemia-guided strategy is chosen for patients at low risk of recurrent ischemia, especially for women. Although PCI is currently the intervention of choice for most of these patients, individual coronary anatomy and clinical features may dictate the use of a different approach, such as coronary artery bypass grafting (CABG), a surgery that reinstates cardiac blood flow (Parsa et al., 2011). In some cases, current guidelines also recommend an antiplatelet therapy combined with non-vitamin K antagonist oral anticoagulant (NOACs) therapy (Husted et al., 2014; Steffel et al., 2018). Indeed, the rates of major complications of acute myocardial infarction have declined dramatically with early reperfusion (PCI) associated with antiplatelet therapy (French et al., 2010). Nevertheless, complications are still a leading cause of death and deserve careful consideration. Several new therapeutic approaches, such as reducing inflammation, mitigating reperfusion injury or inducing myocardial regeneration, are under active investigation although, except for angiotensin converting enzyme (ACE) inhibition, have so far not proved beneficial in the acute care setting. Acute myocardial infarction continues to have a major impact on global health and its management remains a crucial challenge for scientific advancement in medicine (Bhatt et al., 2013).

### Pulmonary Hypertension

Pulmonary hypertension (PH) is a hemodynamic disease state involving multiple clinical conditions including pulmonary arterial hypertension (PAH) and chronic thromboembolic pulmonary hypertension (CTEPH) (Galiè et al., 2016). They are defined by hemodynamic parameters characterized by a mean pulmonary artery pressure ≥ 25 mmHg at rest, measured during right heart catheterization (Galiè et al., 2016). The pathophysiological consequence is a gradual obstruction of the arterial lumen leading to the development of increased resistance of the pulmonary vasculature and, ultimately, right ventricular failure (Galiè et al., 2009; Lang, 2015). PAH is characterized by disease-specific lesions mainly involving the smaller pulmonary arteries (<500 μm in diameter). These lesions feature thickening of both the external and medial layers and changes in the endothelial monolayer accompanied by an inflammatory infiltration and formation of complex thrombotic lesions (Galiè et al., 2009). Unlike PAH, where obstruction caused by remodeling likely occurs in the more distal pulmonary arteries, CTEPH is largely associated with prominent obstructions in the main pulmonary arteries caused by unresolved thrombi affecting the medial and intimal layers of the arteries (Humbert, 2010; Lang, 2015). Subsequently, distal pulmonary arteriopathy and microvascular disease can be triggered, indistinguishable from classic PAH (Lang and Madani, 2014; Lang, 2015). PAH and CTEPH are both rare and progressive vascular diseases with poor prognosis if early diagnosis is not performed. Diagnosis is complicated by the lack of biomarkers and patient-specific symptoms (Humbert et al., 2014). Whereas, PAH has an estimated prevalence of 15 to 50 per million population (Humbert et al., 2014), the prevalence of CTEPH is not easy to estimate, due to a long asymptomatic period between the initiating event (pulmonary embolism; PE) and the overt symptomatic disease. With this caution, the prevalence of CTEPH is estimated at between 0.1 and 9.1% after diagnosis of PE (Humbert, 2010; Lang and Madani, 2014). Current therapies for PAH mainly focus on targeting endothelial dysfunction, whereas pulmonary endarterectomy (PEA) is the treatment of choice for CTEPH, with a possible curative outcome (Nogueira-Ferreira et al., 2014; Jenkins, 2015). To date, additional research is needed to learn more about the onset and development of PH. This review will touch the recent findings concerning the endothelium dysfunction and metabolic alterations in PAH and CTEPH, its likely relationship with the disease pathogenesis, and whether pharmacological modifications on cellular metabolism might be a potential future treatment for PH.

#### Current Therapies in PH

PAH is the most studied condition of all PH clinical groups. Three key vasomotor pathways are the targets of the main approved PAH therapies: (1) prostacyclin, (2) endothelin-1, and (3) nitric oxide-cyclic guanosine monophosphate (cGMP). Interventions on all three pathways aim at restoring the imbalance of endothelial vasodilator and vasoconstrictors mediators. However, because they have little impact on vascular remodeling and coagulation homeostasis, they are not curative for PAH (Humbert et al., 2014).

No targeted therapy is currently available for CTEPH patients and, as mentioned above, pulmonary endarterectomy (PEA) is the treatment of choice in operable patients. Features for determining patient operability are the accessibility of the thromboembolic material and patient comorbidities influencing the peri- and post-operative risk. Effective PEA results in improvement of clinical symptoms, normalization of hemodynamics, and increased survival (Jenkins, 2015). For patients who suffer from inoperable CTEPH or persistent/recurrent CTEPH, Riociguat was recently approved as the only pharmaceutical targeted treatment of CTEPH based on the findings from the CHEST study (Hoeper, 2015) and the PATENT-1 study (Galiè and Ghofrani, 2013). Riociguat is a member of the family of soluble guanylate cyclase stimulators (sGC). It has a two-faceted mode of action, on the one hand by increasing the sensitivity of sGC to endogenous NO, and on the other hand by stimulating sGC activity independently of NO, resulting in restoration of the NO-sGC-cGMP (cyclic guanosine monophosphate) pathway, accompanied with vasodilation and anti-fibrotic, anti-proliferative and anti-inflammatory effects (Hoeper, 2015). The drug significantly improves the patients' exercise capacity and pulmonary vascular resistance and possibly constitutes a first targeted therapy for CTEPH (Ghofrani et al., 2013).

The limited benefits of existing therapies for PAH and the high risk associated with PEA for CTEPH patients have fostered recent studies that explore new therapeutic avenues. An emerging topic suggests the occurrence of potentially actionable metabolic abnormalities in pulmonary hypertension. We will now focus on recent observations on the role of EC dysfunction and metabolism in the pathophysiology of PAH and CTEPH and the therapeutic opportunities they may provide.

## Endothelial Cellular Energy Metabolism

The endothelium is a dynamic organ consisting of a single layer of endothelial cells (ECs) lining the entire vascular system. Independently of their anatomic location (artery, arteriole, capillary, venule, vein) all endothelial share the common function of maintenance of vessel homeostasis (Pober et al., 2009; Sandoo et al., 2010). The control of vessel functions involves regulation of the blood flow, vascular tone, physical barrier, blood coagulation, and the inflammatory response. A balanced production of various hormones, neurotransmitters, and vasoactive factors is crucial for maintaining a homeostatic vessel function (Sandoo et al., 2010). An important vasoactive factor is eNOS-derived nitric oxide (NO), that promotes vasodilatation and inhibits important events that contribute to the development of vascular remodeling diseases, such as platelet aggregation, adhesion of leukocytes, and oxidative stress (Förstermann and Sessa, 2012). NO produced by the endothelium also plays an important role in mitochondrial respiration to maintain the oxygen gradient in oxygen limiting situations (Dromparis and Michelakis, 2013). When the NO precursor arginine and the eNOS cofactor tetrahydrobiopterin (BH_4_) are not available, eNOS fails to produce NO and may promote the formation of reactive oxygen species (ROS), causing endothelial dysfunction and leading to atherosclerosis and PH pathogenesis (Förstermann and Sessa, 2012).

The importance of maintaining physiological and homeostatic EC functions is underlined by the development of major diseases like cardiovascular disease, diabetes, and cancer, consequent to endothelial dysfunction (Goveia et al., 2014). The endothelium can be disrupted by EC damage or apoptosis which leads to a re-endothelialization response and in some cases to the selection of ECs with an altered phenotype (Pober et al., 2009; Nogueira-Ferreira et al., 2014). Environmental stresses, such as oxidative stress and metabolic disturbances, are important sources of endothelial dysfunction, injury, and death (Pober et al., 2009).

### Glycolysis

When exposed to hypoxic conditions, mitochondrial alterations or growth factors, ECs can rapidly shift from a quiescent cellular mode to an angiogenic state accompanied with changes in their cell metabolism. Under basal conditions, ECs rely mostly (>80%) on glycolysis for generating their cellular energy and thus leaving the circulating oxygen available for underlying oxygen-requiring tissues (De Bock et al., 2013b; Dromparis and Michelakis, 2013; Goveia et al., 2014). In pathological conditions with sustained suppressed oxidative phosphorylation (OXPHOS), glycolytic flux is increased regardless of the oxygen supply as long as glucose is available (Warburg effect) promoting the development of highly proliferative vascular disorders (Parra-bonilla et al., 2010; Dromparis and Michelakis, 2013). This predominantly reliance on glycolysis over the use of more efficient mitochondrial oxidation is supported by the presence of fewer mitochondria when compared to cell types relying on mitochondrial respiration. Regardless the presence of sufficient oxygen to maintain mitochondrial respiration, glycolytic flux quickly converts pyruvate to lactate, followed by an increase in glucose uptake (Parra-bonilla et al., 2010). Sustained upregulation of glycolysis and suppression of OXPHOS is often accompanied with a normoxic activation of HIF1α, leading to activation/upregulation of several glycolytic enzymes and further suppressing mitochondrial respiration (Dromparis and Michelakis, 2013).

The glycolytic enzyme phosphofructokinase-2/fructose-2,6-biphosphatase 3 (PFKFB3) is important for maintaining glycolysis and it has been shown that PFKFB3 inactivation reduces EC proliferation (De Bock et al., 2013b). PFKFB3 catalyzes the synthesis of fructose-2,6-biphosphate (Fru-2,6-P_2_), an allosteric activator of phosphofructokinase 1 (PFK-1) and a potent stimulator of glycolysis. Partial reduction of the glycolytic flux by PFKFB3 inhibition was shown to be sufficient to impair EC proliferation without induction of cell death as caused by 2DG (2-deoxy-D-glucose) (Schoors et al., 2014). Another important glycolytic enzyme is the mitochondrial “gate-keeper” enzyme pyruvate dehydrogenase (PDH) that acts as a promoter of the entry of pyruvate in mitochondria (Cottrill and Chan, 2013). PDH can be phosphorylated and inhibited through the activity of pyruvate dehydrogenase kinases (PDKs), resulting in a reduced mitochondrial contribution to energy production and concomitant promotion of aerobic glycolysis (Ryan and Archer, 2015).

PDKs are induced by HIF1α and PDHs are additionally inhibited by altered mitochondrial Ca^2+^ signaling (Dromparis and Michelakis, 2013). Upon PDH inhibition, pyruvate, rather than entering the mitochondrial respiratory chain, is converted to lactate by the enzyme lactate dehydrogenase (LDH) allowing the transformation of NADH to NAD^+^, which is crucial for maintaining further glycolysis. Lactate is released to the extracellular microenvironment via monocarboxylate transporter 4 (MCT4). It can also enter the cells through monocarboxylate transporter 1 (MCT1) and used to feed the TCA cycle under low oxygen conditions, promoting the so-called “reverse Warburg effect,” a process that illustrates the symbiotic relationship between lactate-producing and lactate-consuming normal and pathological cells (Semenza, 2008), an important adaptive mechanism to continuously changing micro-environmental conditions (Semenza, 2008; Draoui and Feron, 2011). Moreover, the increase in lactate production generates an extracellular acidification of the microenvironment which promotes the activity of certain metalloproteases that disrupt the extracellular matrix, which also leads to an infiltration of the vascular wall with inflammatory cells and other cell types (Bonuccelli et al., 2010).

Glycolysis is also connected to the pentose phosphate pathway (PPP), a metabolic pathway that generates NADPH and ribose-5-phosphate, essential for the biosynthesis of lipids and nucleotides. PPP plays a pivotal role in the production of NADPH moieties which provide the reducing equivalents necessary for the synthesis of fatty acids and for the scavenging of ROS to promote cell survival (Patra and Hay, 2014). The altered metabolic profile of mitochondrial suppression along with increased glycolytic rates causing a dysfunctional vasculature is similar to that of other rapidly proliferating healthy and malignant cell types, in which a shift occurs from mitochondrial respiration to lactate-producing aerobic glycolysis in order to sustain their rapid growth and to block apoptosis (Vander Heiden et al., 2009; Parra-bonilla et al., 2010; De Bock et al., 2013a).

### Mitochondrial Respiration

Mitochondria are considered the quintessential cellular engine since its primary function is energy production in the form of adenosine triphosphate (ATP). ATP is essential to sustain cellular bioenergetic demands, glucose being the principal carbon substrate needed to generate ATP. OXPHOS is the mitochondrial metabolic pathway that enables cells to synthesize ATP from oxidation of nutrients. For each glucose molecule that enters the glycolytic pathway, 2 pyruvate molecules are produced with a net energy of 2 ATP molecules. Subsequent pyruvate uptake into mitochondria results in 36 ATP molecules with minimal production of lactate under physiological conditions (Vander Heiden et al., 2009; Parra-bonilla et al., 2010). This process is oxygen-dependent and, considering its high efficiency, it is the predominant source of energy in mammalian cells(Parra-bonilla et al., 2010). Individual cell growth is controlled by environmental nutrient availability; therefore, cells only take up nutrients for cell division when stimulated by growth factors to avoid abnormal proliferation. In addition to ATP, active cell division requires nucleotides, amino acids, and lipids. To maintain cell growth and concomitant increase in biomass, part of the glucose must be redirected to the generation of critical macromolecular precursors such as acetyl-CoA, glycolytic intermediates, and ribose for the biosynthesis of, respectively, fatty acids, non-essential amino acids, and nucleotides (Vander Heiden et al., 2009). In order to promote this flow of carbon substrates toward biomass accumulation, rapidly growing cells are endowed with mechanisms that favor glycolysis over mitochondrial oxidation.

Although the major function of mitochondria is ATP production, these organelles are also important regulators of cell survival, ion homeostasis (H^+^, Ca^2+^) and cellular redox status (Collins et al., 2012). Tight regulation of the mitochondrial ion status is of great importance in tissues with limited oxygen consumption, like the vasculature and the lung, which facilitate the diffusion of oxygen to more oxygen-requiring tissues. Disturbances in mitochondrial ion status have direct and indirect consequences on cell function, growth, and survival (Dromparis and Michelakis, 2013). Altered mitochondrial morphology and function prompted by factors such as NO status have been associated with vascular endothelial dysfunction and to diverse pathological conditions, including cardiovascular disorders, muscular degeneration, and cancer (Collins et al., 2012; Dromparis and Michelakis, 2013). Mitochondrial metabolism contributes actively to the production of reactive oxygen species (ROS). Mitochondria regulate redox signaling to and from mitochondria and initiate cellular apoptosis (Rizzuto et al., 2012). Oxidative stress is considered a major contributor to the destruction of well-balanced homeostatic mechanisms, causing cell injury either through direct oxidation of cellular proteins, lipids, and DNA or via cell death signaling pathways (Leopold and Loscalzo, 2005; Sinha et al., 2014).

The sensitivity of cells to glycolytic and OXPHOS inhibitors (such as2DG, and Oligomycin, respectively) can be used to help unveil the cell dependency on a specific energy-generating pathway (Suganuma et al., 2010). Such studies have shown that, in spite of the importance of the glycolytic pathway, especially under hypoxic conditions, the majority of cells use a combination of OXPHOS and glycolysis as a strategy for energy production, pointing out the importance of metabolic plasticity for cell survival under shifting environments and the complexity of metabolic adaptations in disease (Moreno-Sánchez et al., 2007).

### ROS-Oxidative Stress

During OXPHOS, electrons from NADH and FADH_2_ molecules (electron donors) are transferred to electron acceptors, such as oxygen. These redox reactions are carried out by protein complexes located in the mitochondrial inner membrane, and release energy which is used to form ATP (Vega-Naredo et al., 2014). Small amounts of these electrons form prematurely mitochondria-derived reactive oxygen species (mROS), such as superoxide (O${}_{2}^{-}$). ROS are oxygen-containing chemically reactive species which play important signaling roles to sustain fundamental cellular functions under various physiological conditions (Förstermann, 2008). High levels of oxygen and increased mitochondrial activity leads to excessive ROS production overcoming the buffer capacity of usable antioxidant systems results in oxidative stress causing increased cell death and endothelial dysfunction (Leopold and Loscalzo, 2005; Pangare and Makino, 2012; Dromparis and Michelakis, 2013; Li et al., 2014). Mitochondria-based manganese superoxide dismutase (MnSOD, or SOD2) has an immediately anti-oxidative effect through the conversion of superoxide to the more stable and diffusible H_2_O_2_. After leaving mitochondria, physiological levels of hydrogen peroxide (H_2_O_2_) function as an important signaling system, acting on several redox sensitive targets in the cytoplasm (HIF1α) and the cell membrane (K^+^ channels) and also with some proliferation-related enzymes (Fukai and Ushio-Fukai, 2011; Dromparis and Michelakis, 2013). Several other enzyme systems can produce ROS in the vascular wall, notably NADPH oxidase, xanthine oxidase, and eNOS, a dysfunctional endothelial NO synthase in which oxygen reduction is uncoupled from NO synthesis (Brandes and Kreuzer, 2005; Förstermann, 2008; Li et al., 2014; Xia et al., 2017). These oxidases are multisubunit enzyme complexes that produce superoxide from molecular oxygen and NADPH as electron donor (Drummond et al., 2011).

Imbalances in the cellular oxidative status play significant roles in the pathophysiology of vascular diseases (Santos et al., 2011). Some cells might resort to a metabolic switch to glycolysis as a mechanism to reduce production of ROS and thus to protect themselves from mitochondrial-mediated apoptosis (Ryan and Archer, 2015). The crucial role of oxidative stress in CVDs makes it an attractive target for therapy. However, recent studies in patients with cardiovascular symptoms showed that the use of supplementary antioxidants such as vitamins E and C had little therapeutic effect. This was mainly due to the limited specificity for ROS producing factors and to the requirement of high antioxidant doses which could worsen vascular function (Münzel et al., 2010, 2017). Whilst, novel therapeutic strategies propose to target more precise ROS producing sites such as the mitochondria, it is not an easy task as those are important dose-dependent signaling molecules which could have a detrimental effect on key body functions. Therefore, more detailed studies are needed to elucidate the potential therapeutic effects of antioxidant treatments(Münzel et al., 2017).

## The Importance of the Endothelium in Disease

### Endothelial Dysfunction in Acute Myocardial Infarction

**Figure 1:**
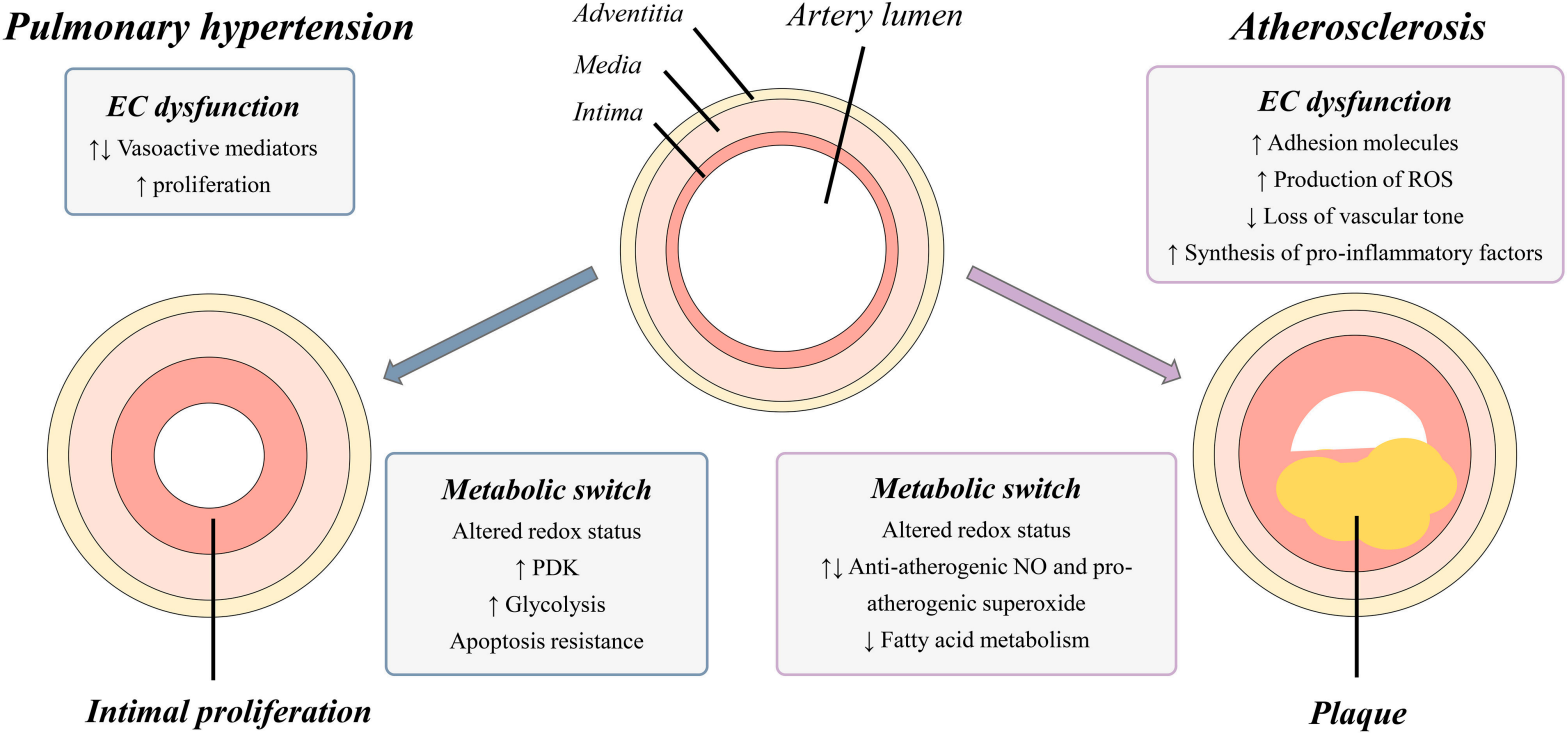
Graphical representation of vascular changes occurring in PH and atherosclerosis. Narrowing of the artery lumen is caused by intimal proliferation (PH) and by plaque formation (atherosclerosis) and is a result of disease specific EC dysfunction and cellular metabolic switches. EC, endothelial cell; PDK, pyruvate dehydrogenase kinases; ROS, reactive oxygen species; NO, nitric oxide; PH, in pulmonary hypertension.

Acute myocardial infarction (AMI) is the development of myocardial necrosis caused by an unstable ischemic state. The disorder is diagnosed and assessed based on clinical evaluation, electrocardiogram (ECG), biochemical testing, invasive and non-invasive imaging, and pathological evaluation. The usual triggering event of acute myocardial infarction is the rupture or erosion of a vulnerable, lipid-laden, atherosclerotic coronary plaque. This event results in the exposure of circulating blood to highly thrombogenic core and matrix materials in the plaque (Badimon et al., 2012). In response to various stimuli, the normal endothelium endures phenotypic changes and variations, collectively known as endothelial dysfunction, characterized by a loss of the majority of the homeostatic mechanisms present in normal healthy endothelial cells. Usually, this dysfunction is associated with upregulation of adhesion molecules, enhanced production of ROS, synthesis of pro-inflammatory factors and loss of vascular tone (Figure 1) (Sitia et al., 2010). Recent studies suggest that this dysfunction contributes to the progression of the atherosclerotic plaque (Zardi and Afeltra, 2010). The pioneer role played by endothelial dysfunction in vascular pathology is supported by observations that individuals without any clinical sign of atherosclerosis but with high cardiovascular risk already present an endothelial dysfunction indicated by a diminished response to some vasodilators, such as acetylcholine (Zardi and Afeltra, 2010). These findings suggest that endothelial dysfunction may precede and constitute a link between different vascular diseases and represents a good predictor of future cardiovascular events, including atherosclerotic diseases and AMI (Sitia et al., 2010). Endothelial dysfunction is considered a systemic vascular process that not only leads to plaque formation, but also determines the clinical course of atherosclerosis progression and associated coronary syndromes. Because several metabolic pathway abnormalities, such as the deregulation of the nitric oxide production and the excessive generation of ROS (Table 1), are associated with atherosclerosis, the identification of key metabolic mechanisms underlying such alterations should provide fresh opportunities for the development of new strategies for the treatment of endothelial cell dysfunction in atherosclerosis and related vascular pathologies (Bierhansl et al., 2017).

**Table 1:** Summary main metabolic perturbations in AMI and PH.

	**AMI**	**Pulmonary Hypertension**
Nitric Oxide	↓ NO^a^, ^b^	↓ NO^d^
Proliferation	↓ proliferation^c^	↑ proliferation^e^
Metabolism	↓Glycolysis^a^	↓ OXPHOS ↑ Glycolysis^f^
Antioxidants and ROS	↑ mROS^a^, ^b^	↓MnROS (↑O${}_{2}^{-}$/↓H_2_O_2_)^g^

a Eelen et al., 2015;

b Förstermann and Sessa, 2012;

c Lu et al., 2013;

d Budhiraja et al., 2004;

e Humbert et al., 2004; Masri et al., 2007;

f Xu et al., 2007; Xu and Erzurum, 2011; Lewis, 2014; Bujak et al., 2016;

g *Archer et al., 2008*.

### Metabolic Alterations and Requirements in Acute Myocardial Infarction

Atherosclerosis is characterized by the presence of an uncoupled and reduced eNOS, causing an imbalance between the production of NO, an anti-atherogenic molecule, and superoxide, a pro-atherogenic factor, thereby losing the atheroprotective function of eNOS (Eelen et al., 2015). As such, two general processes are largely responsible for the angiogenic growth observed in early atherogenesis: inflammation and oxidative stress. These metabolic alterations also affect, to a variable degree, vascular remodeling and coagulation homeostasis (Humbert et al., 2014). Beyond eNOS, a critical role in atherogenesis is also played by NADPH oxidase (NOX) enzymes, a large family of enzymes that are pivotal in the generation of ROS in the vasculature. NOX-4 is universally expressed in vascular smooth muscle cells (VSMCs), the primary components of the vascular wall and crucial determinants of vascular homeostasis and disease (Lu et al., 2013). Its expression and activation during the angiogenic process promotes a chain of events leading to vascular inflammation, cellular dysfunction, and atherosclerosis.

Additional metabolic mechanisms may contribute to the generation of a pro-inflammatory environment leading to atherogenesis. For example, the high glycolytic mode encountered in endothelial dysfunction implies a relative reduction in available ATP as compared to cells with OXPHOS-predominant metabolism. This results in diminished intracellular adenosine levels which drives hydrolysis of S-adenosylhomocysteine (SAH) to adenosine and L-homocysteine (Hcy). Reduced SAH levels foster histone H3 lysine 4 hypomethylation and overexpression of a pro-inflammatory gene repertoire (Xu et al., 2017). A recent study (Ward-Caviness et al., 2017) identified three metabolic biomarkers, arginine and two lysophosphatidylcholines (LPC 17:0 and LPC 18:2) associated with incident myocardial infarction (MI). This study also focus on the association between these metabolites and the high-sensitivity C reactive protein (hsCRP), which is a measure for inflammation (Kaptoge et al., 2012). The three biomarkers correlated with each other and with the hsCRP levels, suggesting that inflammation can represent a pathway through which these biomarkers are associated with MI (Cheng et al., 2012). As a consequence of these processes, increased rates of apoptosis are evident in more advanced atherosclerotic plaques. This is a key point in the progression of atherosclerosis and consists of a programmed cell death, morphologically expressed as cellular contraction, condensation of chromatin, and disruption of the cellular membrane. All cells existing in the atherosclerotic plaque, including lymphocytes, endothelial cells, smooth muscle cells, and macrophages, can undergo cellular apoptosis (Schrijvers et al., 2005).

#### Future Treatment Options in AMI

Currently, the use of beta blockers and angiotensin-converting enzyme (ACE)-inhibitors are under study for the treatment of AMI, as well as improvements in antithrombotic therapies. Despite the advances of the last decades, a lot is unknown about the biological processes involved in cardiac development and repair for this reason there is a strong need to find new successful therapeutic targets to struggle this disease (Ibanez et al., 2018).

Metabolomic approaches can identify potential biomarkers with predictive value for CVD (Hoefer et al., 2015) and correlate metabolic profiles with risk of death or incident MI (Shah et al., 2010). Several studies focus on the limitations of current vascular biomarkers, like hsCRPs (high sensitive C reactive proteins) and encourage the discovery and validation of novel biomarkers using emerging omics technologies. HDL cholesterol is considered strictly linked to cardiovascular diseases: low HDL cholesterol is associated to a very high cardiovascular risk while high HDL cholesterol seems to be linked to cardiovascular protection (Rohatgi et al., 2014). It has been described, that the key role of HDL is to promote the efflux of the cholesterol and to invert its transport from the periphery to the liver, which seems to be correlated to a low incidence of cardiovascular events, suggesting the potential use of HDL as a new biomarker for coronary heart disease (Hoefer et al., 2015).

The detection of plasma-derived markers which include microparticles (MPs), microvesicles and exosomes in human plasma, has triggered increasing interest for their potential as biomarkers. In particular, levels of MPs expressing CD31 or CD144 seem inversely correlated to the endothelium associated vasodilation highlighting that MPs' levels might be good indicators of vascular lesions and acute endothelial dysfunction (Lacroix et al., 2010). Moreover, the complex composition of these microparticles which comprise proteins, lipids, and nucleic acids represent an interesting onset for omics based analysis (Kanhai et al., 2013). Recent studies have also unveiled that micro-RNAs (miRNAs) not only present relevant intracellular functions, but also show potential value as cardiovascular disease biomarkers. miRNAs also circulate within microvesiscles and may contribute to forecast heart failure, early atherosclerosis and plaque vulnerability by targeting vascular and cardiac cells (Matsumoto et al., 2013).

Metabolomic studies using general population-based cohorts have recently been performed using LC-MS/MS-based lipidomics and NMR-based approaches to identify species associated with incident CVD with a potential link to systemic inflammation and in particular, pro-inflammatory lipid metabolites have acquired great interest in the cardiovascular disease frame (Hoefer et al., 2015). Molecular lipid profiling by mass spectrometry and nuclear magnetic resonance spectroscopy, as proteomic identification and quantification of small metabolites, can improve the individual cardiovascular risk prediction (Stegemann et al., 2014).

The identification of potential metabolic targets for novel therapeutic approaches for acute myocardial infarction is therefore an interesting approach that should be investigated in earnest in the near future.

### Endothelial Dysfunction in Pulmonary Hypertension

The pulmonary vascular barrier consists of three cellular layers: an external layer, the adventitia containing fibroblasts, a medial layer mainly consisting of smooth muscle cells (SMC) lined by an elastic membrane and an internal intima composed of a single layer of endothelial cells in direct contact with the blood circulation (Jeffery and Morrell, 2002). Upon abnormal activation, the normally quiescent endothelium loses its homeostatic function, leading to a disorganization of the three-layer structure of the vascular wall as a key element in the development of pathological lesions (Budhiraja et al., 2004; Xu and Erzurum, 2011). Several endothelium activation stimuli such as ROS, shear stress and inflammation are known to stimulate the endothelium, causing changes in its proliferation status and production of vasoactive mediators and growth factors. In PH, it has been shown that there is an imbalance between vasodilators, such as nitric oxide (NO) and prostacyclin (PG), and vasoconstrictors, such as endothelin-1 (ET-1) and thromboxane, resulting in the disruption of basal pulmonary vascular tone, vascular injury repair and growth (Budhiraja et al., 2004). In addition to changes in vasoactive mediators, it is hypothesized that initial endothelial damage induces a widespread endothelial cell death cascade leading to an apoptosis-resistant population, from which rapidly proliferating cells regenerate the vascular lining (Figure 1) (Masri et al., 2007). Additionally, endothelium injury also causes exposure of the medial and adventitial layer to growth factors, inducing the proliferation of fibroblasts and smooth muscle cells (Budhiraja et al., 2004). Furthermore, disease-related alterations in the function and expression of specific ion channels in pulmonary artery smooth muscle cells (PASMC) and endothelial cells contribute to increased vascular tone, proliferation., and decreased apoptosis in PAH. Well-studied as important contributors to continuous vasoconstriction and remodeling of pulmonary arteries are dysfunctional K^+^ channels and altered levels of cytosolic Ca^2+^(Makino et al., 2017). Regulatory dysfunctions in fibroblasts are an additional factor leading to impaired vascular function and remodeling. Studies in PAH have shown a rise in the deposition of extracellular matrix (ECM) proteins in the adventitia facilitating the migration of fibroblasts to the medial and endothelium layers (Stenmark et al., 2006). The overall result is the emergence of an aberrant apoptosis-resistant, highly proliferative pulmonary vascular endothelium together with disruptions in both fibroblasts and PASMC layers.

Previous research has suggested that PAH ECs derived from obstructive plexiform lesions are likely associated with alterations of cellular functions involved in apoptotic and proliferative processes (Humbert et al., 2004). PAH ECs display faster growth rates as compared to non-diseased pulmonary ECs, as shown by enhanced cellular survival signals and increased cell division. This increased ability to maintain cell viability is accompanied with an upregulation of pro-survival factors and continuous activation of signal transducers and activators of transcription (STATs), such as STAT3, with a known involvement in cell growth regulation (Masri et al., 2007).

In CTEPH, several studies have highlighted the existence of an altered cellular phenotype in cells derived from large arteries. Pulmonary arterial ECs (PAECs) derived from CTEPH patients showed enhanced mitogenic activity *in vitro* (Quarck et al., 2012). This was concomitant with the presence of small-vessel abnormalities such as thickening of the medial layer and increased proliferative characteristics of cells lining the internal intimal layer, and formation of obstructive plexiform lesions comparable with features seen in PAH, which suggests the possibility that both diseases might develop from a common endothelial dysfunction that contribute to vascular remodeling (Piazza and Goldhaber, 2011). A deeper understanding of the cellular processes behind these endothelial abnormalities might therefore shed light on the precise mechanisms that underlie the pathological changes occurring in the vascular wall of PAH and CTEPH.

### Metabolic Alterations and Requirements in Pulmonary Hypertension

To better understand pulmonary vascular remodeling processes, we will take a closer look at the metabolic alterations and requirements of ECs in PH. As described above, endothelial cells are highly dynamic and rely mostly on glycolysis for their energy production and, when stimulated, they further boost the glycolytic rate to support their higher growth rates.

Both *in vitro* and *in vivo* studies have described an increase in glycolytic rate and lactate release in PAECs derived from PAH patients, as compared to non-diseased PAECs (Xu et al., 2007; Xu and Erzurum, 2011). These findings suggest that glucose metabolism is the primary energy source in PAEC. Additionally, PAH PAEC showed decreased oxygen consumption and maintained similar ATP levels under normoxia and hypoxia, compared to control PAECs (Xu et al., 2007). Despite a significant scientific effort in the past years, we are still not able to fully understand regulatory mechanisms that promote the switch from oxidative glucose metabolism to glycolysis. A possible explanation is an impaired mitochondrial function, including pathological activation of pyruvate dehydrogenase kinase (PDK) activity and MnSOD deficiency (Archer et al., 2008; Hernandez-saavedra et al., 2017). It has been shown that pyruvate dehydrogenase kinases (PDK) are highly expressed in PAH, which may imply a stronger inhibition of PDH and thus a proneness toward aerobic glycolysis (Cottrill and Chan, 2013; Ryan and Archer, 2015). Reduced levels of MnSOD in PAH disturb the cellular redox status leading to an accumulation of superoxide anion O${}_{2}^{-}$ and a reduced production of signaling moleculeH_2_O_2_ followed by normoxic activation of the redox-sensitive HIF1α. This pseudohypoxic state, decreased MnSOD and increased PDK in the presence of normal PO_2_, further favors glycolysis and causes a cascade of downstream pathways promoting proliferation and inhibiting apoptosis trough increased cytosolic Ca^2+^ and K^+^ concentrations, respectively, both induced by a downregulation of K_v1, 5_ channel (Archer et al., 2008).

Recent metabolic studies focus on the less invasive technique, metabolomics of biofluids in PH. Despite contrasting reports using this approach regarding PAH (Zhao et al., 2014; Bujak et al., 2016), it is a promising technique in search of disease specific biomarkers. Metabolic profiling in PH has complement findings from *in vitro* and *in vivo* studies regarding increased glycolysis and has additionally found an increase in PPP, decrease in fatty acid oxidation (FAO) and impaired TCA (Lewis, 2014; Bujak et al., 2016). All these observations point to similarities in metabolic profiles between diseased endothelia in PAH and rapidly growing cells, thus suggesting the existence of a Warburg effect in PAH PAECs together with the presence of mitochondrial abnormalities as summarized in Table 1. It will be interesting to determine whether CTEPH ECs also present similar pathophysiological metabolic processes.

#### Future Treatment Options in PAH and CTEPH

The above described metabolic transformations in PAH ECs (enhanced glycolytic flows and diminished oxidative metabolism) bear similarities to the metabolic profiles of hyperproliferative ECs. On that basis, pharmacological blockade of PFKFB3, which restraints angiogenesis (Schoors et al., 2014), offers a window of opportunity to rein in the hyperproliferative state in PAH ECs by reducing their glycolytic rate (Goveia et al., 2014). *In vitro* studies indicated that a dose-dependent inhibition of PFKFB3 by small compounds such as 3PO (3-(3-pyridinyl)-1-(4-pyridinyl)-2-propen-1-one) successfully reduced glycolysis partially (35–40%) although sufficiently to impair EC proliferation (Schoors et al., 2014). The enhanced expression of the PDK enzymes in PAH and CTEPH ECs offers an additionally opportunity for new targeted therapies. Preclinical intervention models of pulmonary hypertension have been studied to evaluate the efficacy of PDK inhibition in limiting and/or overturning pulmonary vascular changes (Harvey and Chan, 2017). The small molecule dichloroacetate (DCA), a pyruvate analog, has a relatively high specific binding to PDK (McMurtry, 2004). In rat models of monocrotaline-induced PAH (MCT-PAH), DCA was shown to prevent and reverse pulmonary vascular remodeling. Further, DCA induced apoptosis in the MCT-PAH pulmonary arteries (PAs) and suppressed cell growth rates measured by bromodeoxyuridine (BrdU) uptake in the medial layer of remodeled MCT-PAH PAs with little impact on normal pulmonary vascular cells in rats and humans, making it a promising small molecule for targeted restoration of normal metabolic dysfunctions in PAH ECs (Stacpoole et al., 2003; McMurtry, 2004; Ryan and Archer, 2015). A recently published work demonstrated in a first-in-human clinical trial that PDK inhibition has a positive effect on the hemodynamics of PAH patients, further supporting the potential of DCA as a pharmacological agent in this disease (Michelakis et al., 2017). Growing evidence of the existence of metabolic remodeling beyond the glycolytic pathway leads to a growing list of possible metabolic targets. One of these targets is the methylation of SOD2 (MnSOD) which suppresses expression of this redox enzyme. 5-AZA (5-aza-2′-deoxycytidine) inhibits methylation of SOD2 followed by restoration of the mitochondrial respiration (Harvey and Chan, 2017). Nevertheless, future metabolic studies in larger PAH patients groups and CTEPH patients will be as necessary as additional steps in unraveling further pathophysiological mechanisms. However, one needs to bear in mind the challenges of metabolic therapies linked to interpatient variations and target selectivity but also due to the complexity of cell metabolism itself. Together with the search for new pharmacological interventions, a broad metabolomic screening is indispensable in the search for biomarkers in PAH and CTEPH that may help early diagnosis and uncover metabolic-mediated remodeling in PH.

## Conclusions and Prospects

There is increasing evidence that endothelial dysfunction in cardiopulmonary vascular disorders is associated with disease-specific metabolic alterations in endothelial cells. Such reorganization of metabolic networks is a double-edged sword as, on the one hand, it can function as a defense mechanism against disease-associated external insults, such as changes in glucose or oxygen availability, while, on the other hand, it can contribute to the generation of toxic end-products, anomalous accumulation of metabolic intermediates and alterations in energetic and redox metabolism that compromise physiological endothelial functions. This review points out current metabolic changes that have a great impact on the onset and progression of both atherosclerosis and PH. Endothelial cells' ability to easily switch between glycolysis, OXPHOS, PPP, and FAO makes the cellular metabolic switch a complex and challenging target in the search for future pharmacological interventions.

Targeting endothelial cell metabolism is a promising strategy to restore normal endothelial function and it has been reported that moderate inhibition of PFKFB3 can block pathological angiogenesis and normalize EC dysfunction, whereas strong inhibition can result in vessel disintegration (Conradi et al., 2017). This strategy could be useful to restore endothelial cell function in pulmonary arterial hypertension (PAH), and possible also in CTEPH, both characterized by abnormal growth and enhanced glycolysis of pulmonary artery endothelial cells (Caruso et al., 2017). The main metabolic perturbations in pathological ECs in atherosclerosis, acute myocardial infarction and PH impact distinct pathways that lead to an imbalance in NO metabolism and ROS production. These diverse metabolic alterations in different EC dysfunctional pathologies highlight the need to apply metabolic network modeling approaches to identify key players that may be specific of endothelial metabolic dysfunctions and to rationally design interventions to target pathological EC metabolism for therapeutic benefit. Although the key players in glycolysis and energetic metabolism as well as in NO and ROS balance have been described, and communalities among different cell types and tissues have been reported, we still have only partial knowledge on the weight of each enzyme on the metabolic network fluxes in different cell types or in pathological vs. healthy states. Techniques and algorithms developed in the past few years permute an accurate modeling of metabolic network fluxes that, in conjunction with “omics” approaches, lay the foundations for unbiased and large-scale identification of targets with the greatest potential for specifically modulating metabolic pathway flux in disease conditions.

For medium-scale metabolic network models, among the main approaches used to model metabolic pathways are those based on the use of stable-isotope tracers (such as ^13^C labeled metabolites). Computational tools to reconstruct metabolic flux maps from the quantification of the incorporation of ^13^C-atoms into metabolites have been developed in recent years (e.g., Young, 2014; Antoniewicz, 2015; Foguet et al., 2016).

Models considering the kinetic properties of each enzyme in the metabolic network, in combination with methodologies such as Metabolic Control Analysis (MCA), a methodology to quantitatively evaluate the relative contribution and importance of each metabolic step in controlling overall metabolic flux distribution, have also been successfully used to predict putative drug targets (Hornberg et al., 2007). The control that each individual enzyme has on the flux through a metabolic pathway is quantified using MCA methodology in terms of the so-called “flux control coefficients.” MCA can be used also to compare metabolic flux distribution in healthy vs. pathological condition. These comparisons will permit to identify the steps with the greatest potential for target specific metabolic adaptations accompanying pathological states and restore the flux distribution in health condition.

For large-scale models, constraint-based genome-scale metabolic models (GSMMs) have been developed and used in the last years to successfully predict putative therapeutic targets in different types of cancer (Ryu et al., 2015; Nilsson and Nielsen, 2016; Zhang and Hua, 2016; Yilmaz and Walhout, 2017). In brief, GSMMs mainly use transcriptomics and other “omics” data to constraint metabolic flux maps as well as flux balance analysis (FBA) methods to optimize for a cellular objective function. The main challenge in this approach is to appropriately define the objective function. To identify drug targets that impact abnormal endothelial cell growth, the most used objective function has been the maximization of biomass production. Using this approach, putative drug targets have been identified relevant to cancer through a systematic search for essential genes and combinations of target genes interacting in a synthetic lethal fashion able to impair biomass production.

As a word of caution, although topology, stoichiometry and chemical reaction properties are the major constraints on metabolic network flux, the consequences of inhibiting an enzyme activity on the overall network flux redistribution will depend on the relative concentrations of the different enzymes in the network (Cascante et al., 2002; De Atauri et al., 2011; Kell and Goodacre, 2014). Relative levels of enzyme concentrations in human metabolic networks are different not only in different tissues and in health vs. disease conditions but also between individuals which can result in different patient responses to identical metabolic drug interventions. Endothelial cell metabolic alterations associated with cardiopulmonary vascular disorders have been mainly studied using immortalized cell models and there is a shortage of studies characterizing ECs from patient tissues, a necessary next step forward in the field.

The therapeutic possibilities of targeting EC metabolism to improve cardiopulmonary vascular dysfunction are still understudied. However, we predict that deeper characterization of metabolic reprogramming in patient-derived cells and systemic approaches that integrate “omics” data into comprehensive metabolic network flux models will soon permit to identify putative key players in endothelial dysfunction associated with cardiovascular disorders and to design personalized multi-hit interventions at the metabolic level to restore physiological endothelial functions.

Finally, growing interest has focused on the modulation of gut microbiota as a therapeutic strategy in cardiovascular diseases. Microbiota stability is essential in human physiology, as it is involved in the regulation of many host functions such as blood pressure control, glucose tolerance, insulin sensitivity, and body weight control among others. Since hypertension and metabolic disturbances are well-known risk factors of CVD development, it is inevitable to touch in this review the possible role of gut microbiota and its impact on the cardiovascular system (Serino et al., 2014; Tang et al., 2017). In humans, the bacterial proportion of the gut microbiota consists of mainly Firmicutes and Bacteroidetes phyla and its ratio is an important indicator of microbiota stability (Tang et al., 2017). Since gut microbes primarily use ingested nutrients as fuel, it is not surprising that changes in dietary patterns alters the gut composition and its functions (Gentile and Weir, 2018). One way of interaction between the gut microbiota and the host is through production of metabolites that are biologically active or further metabolized by the host (Tang et al., 2017). A meaningful example is trimethylamine (TMA), a metabolite produced by Firmicutes phyla that promotes foam cell formation through its hepatic oxidized form TMA-N-oxide (TMAO) (Serino et al., 2014). Increased levels of this atherogenic microbial metabolite have been associated with increased risks of cardiovascular events (Serino et al., 2014; Tang et al., 2017). Individual differences in the composition of the gut bacteria, combined to the plasticity of the microbiota, indicate that a gut microbiota-targeted strategy could be a promising approach for the prevention and the treatment of several metabolic diseases. Despite, to date, little evidence have provided direct evidence of mechanistic or causal roles of gut microbiota in human cardiovascular disease suggesting that the relationship between human and gut microbiota must be further investigated (Griffin et al., 2017; Gentile and Weir, 2018).

## Author Contributions

VS, EZ, OT-C and MCas conceived, designed, and wrote the manuscript. PQ, MCar, JB, and TT contributed to the writing of the manuscript and revised the article critically for significant intellectual content. VS and EZ equally contributed to this work.

### Conflict of Interest Statement

The authors declare that the research was conducted in the absence of any commercial or financial relationships that could be construed as a potential conflict of interest.

## References

[B1] Adams S. P. Sekhon S. S. Wright J. M. (2014). Rosuvastatin for lowering lipids (review). Cochrane Database Syst. Rev. 11, 1–262. 10.1002/14651858.CD010254.pub2 PMC6463960 25415541

[B2] Ahmad F. Chung Y. W. Tang Y. Hockman S. C. Liu S. Khan Y.. (2016). Phosphodiesterase 3B (PDE3B) regulates NLRP3 inflammasome in adipose tissue. Sci. Rep. 6, 1–13. 10.1038/srep28056 27321128 PMC4913246

[B3] Anderson J. L. Morrow D. A. (2017). Acute myocardial infarction. N. Engl. J. Med. 376, 2053–2064. 10.1056/NEJMra1606915 28538121

[B4] Antoniewicz M. R. (2015). Methods and advances in metabolic flux analysis: a mini-review. J. Ind. Microbiol. Biotechnol. 42, 317–325. 10.1007/s10295-015-1585-x 25613286

[B5] Archer S. L. Gomberg-maitland M. Maitland M. L. Rich S. Garcia J. G. Weir E. K. (2008). Mitochondrial metabolism, redox signaling, and fusion : a mitochondria-ROS-HIF-1-Kv1. 5 O2-sensing pathway at the intersection of pulmonary hypertension and cancer. Am. J. Physiol. Hear. Circ. Physiol. 294, 570–578. 10.1152/ajpheart.01324.2007 18083891

[B6] Badimon L. Padró T. Vilahur G. (2012). Atherosclerosis, platelets and thrombosis in acute ischaemic heart disease. Eur. Hear. J. Acute Cardiovasc. Care 1, 60–74. 10.1177/2048872612441582 24062891 PMC3760546

[B7] Bergheanu S. C. Bodde M. C. Jukema J. W. (2017). Pathophysiology and treatment of atherosclerosis: current view and future perspective on lipoprotein modification treatment. Netherlands Hear. J. 25, 231–242. 10.1007/s12471-017-0959-2 28194698 PMC5355390

[B8] Bhatt D. L. Stone G. W. Mahaffey K. W. Gibson C. M. (2013). Effect of platelet inhibition with cangrelor during PCI on ischemic events. N. Engl. J. Med. 368, 1303–1313. 10.1056/NEJMoa1300815 23473369

[B9] Bierhansl L. Conradi L. C. Treps L. Dewerchin M. Carmeliet P. (2017). Central role of metabolism in endothelial cell function and vascular disease. Physiology 32, 126–140. 10.1152/physiol.00031.2016 28202623 PMC5337830

[B10] Bonuccelli G. Tsirigos A. Whitaker-Menezes D. Pavlides S. Pestell R. G. Chiavarina B.. (2010). Ketones and lactate “fuel” tumor growth and metastasis: evidence that epithelial cancer cells use oxidative mitochondrial metabolism. Cell Cycle 9, 3506–3514. 10.4161/cc.9.17.12731 20818174 PMC3047616

[B11] Brandes R. P. Kreuzer J. (2005). Vascular NADPH oxidases: molecular mechanisms of activation. Cardiovasc. Res. 65, 16–27. 10.1016/j.cardiores.2004.08.007 15621030

[B12] Budhiraja R. Tuder R. M. Hassoun P. M. (2004). Endothelial dysfunction in pulmonary hypertension. Circulation 109, 159–165. 10.1161/01.CIR.0000102381.57477.50 14734504

[B13] Bujak R. Mateo J. Blanco I. Izquierdo-García J. L. Dudzik D. Markuszewski M. J.. (2016). New biochemical insights into the mechanisms of pulmonary arterial hypertension in humans. PLoS ONE 11:e0160505. 10.1371/journal.pone.0160505 27486806 PMC4972307

[B14] Caruso P. Dunmore B. J. Schlosser K. Schoors S. Dos Santos C. Perez-Iratxeta C. (2017). Identification of miR-124 as a major regulator of enhanced endothelial cell glycolysis in pulmonary arterial hypertension via PTBP1 and PKM2. Circulation 136, 2451–2467. 10.1161/CIRCULATIONAHA.117.028034 28971999 PMC5736425

[B15] Cascante M. Boros L. G. Comin-Anduix B. de Atauri P. Centelles J. J. Lee P. W. (2002). Metabolic control analysis in drug discovery and disease. Nat. Biotechnol. 20, 243–249. 10.1038/nbt0302-243 11875424

[B16] Catapano A. L. Graham I. De Backer G. Wiklund O. Chapman M. J. Drexel H.. (2016). 2016 ESC/EAS Guidelines for the management of dyslipidaemias. Eur. Heart J. 37, 2999–3058l. 10.1093/eurheartj/ehw272 27567407

[B17] Cheng S. Rhee E. P. Larson M. G. Lewis G. D. McCabe E. L. Shen D. (2012). Metabolite profiling identifies pathways associated with metabolic risk in humans. Circulation 125, 2222–2231. 10.1007/978-1-62703-673-3 22496159 PMC3376658

[B18] Cohen J. C. Boerwinkle E. Mosley T. H. Hobbs H. H. (2006). Sequence variations in PCSK9, low LDL, and protection against coronary heart disease. N. Engl. J. Med. 354, 1264–1272. 10.1056/NEJMoa054013 16554528

[B19] Collins Y. Chouchani E. T. James A. M. Menger K. E. Cochemé H. M. Murphy M. P. (2012). Mitochondrial redox signalling at a glance. J. Cell Sci. 125(Pt 4):801–806. 10.1242/jcs.110486 22448036

[B20] Conradi L. C. Brajic A. Cantelmo A. R. Bouché A. Kalucka J. Pircher A.. (2017). Tumor vessel disintegration by maximum tolerable PFKFB3 blockade. Angiogenesis 20, 599–613. 10.1007/s10456-017-9573-6 28875379

[B21] Cottrill K. A. Chan S. Y. (2013). Metabolic dysfunction in pulmonary hypertension: the expanding relevance of the warburg effect. Eur. J. Clin. Invest. 43, 855–865. 10.1126/scisignal.2001449 23617881 PMC3736346

[B22] de Atauri P. Benito A. Vizán P. Zanuy M. Mangues R. Marín S.. (2011). Carbon metabolism and the sign of control coefficients in metabolic adaptations underlying K-ras transformation. Biochim. Biophys. Acta Bioenerg. 1807, 746–754. 10.1016/j.bbabio.2010.11.015 21185256

[B23] De Bock K. Georgiadou M. Carmeliet P. (2013a). Role of endothelial cell metabolism in vessel sprouting. Cell Metab. 18, 634–647. 10.1016/j.cmet.2013.08.001 23973331

[B24] De Bock K. Georgiadou M. Schoors S. Kuchnio A. Wong B. W. Cantelmo A. R.. (2013b). Role of PFKFB3-driven glycolysis in vessel sprouting. Cell 154, 651–663. 10.1016/j.cell.2013.06.037 23911327

[B25] Dewey F. E. Gusarova V. O'Dushlaine C. Gottesman O. (2017). Inactivating variants in ANGPTL4 and risk of coronary artery disease. N. Engl. J. Med. 374, 1123–1133. 10.1056/NEJMoa1510926 26933753 PMC4900689

[B26] Draoui N. Feron O. (2011). Lactate shuttles at a glance: from physiological paradigms to anti-cancer treatments. Dis. Model. Mech. 4, 727–732. 10.1242/dmm.007724 22065843 PMC3209642

[B27] Dromparis P. Michelakis E. D. (2013). Mitochondria in vascular health and disease. Annu. Rev. Physiol. 75, 95–126. 10.1146/annurev-physiol-030212-183804 23157555

[B28] Drummond G. R. Selemidis S. Griendling K. K. Sobey C. G. (2011). Combating oxidative stress in vascular disease: NADPH oxidases as therapeutic targets. Nat. Rev. Drug Discov. 10, 453–471. 10.1038/nrd3403 21629295 PMC3361719

[B29] Eelen G. de Zeeuw P. Simons M. Carmeliet P. (2015). Endothelial cell metabolism in normal and diseased vasculature. Circ. Res. 116, 1231–1244. 10.1161/CIRCRESAHA.116.302855 25814684 PMC4380230

[B30] Foguet C. Marin S. Selivanov V. A. Fanchon E. Lee W. N. Guinovart J. J.. (2016). HepatoDyn: a dynamic model of hepatocyte metabolism that integrates 13C isotopomer data. PLoS Comput. Biol. 12:e1004899. 10.1371/journal.pcbi.1004899 27124774 PMC4849781

[B31] Förstermann U. (2008). Oxidative stress in vascular disease: causes, defense mechanisms and potential therapies. Nat. Clin. Pract. Cardiovasc. Med. 5, 338–349. 10.1038/ncpcardio1211 18461048

[B32] Förstermann U. Sessa W. C. (2012). Nitric oxide synthases: regulation and function. Eur. Heart J. 33, 829–837. 10.1093/eurheartj/ehr304 21890489 PMC3345541

[B33] French J. K. Hellkamp A. S. Armstrong P. W. Cohen E. Kleiman N. S. O'Connor C. M.. (2010). Mechanical complications after percutaneous coronary intervention in ST-elevation myocardial infarction (from APEX-AMI). Am. J. Cardiol. 105, 59–63. 10.1016/j.amjcard.2009.08.653 20102891

[B34] Fukai T. Ushio-Fukai M. (2011). Superoxide dismutases: role in redox signaling, vascular function, and diseases. Antioxid. Redox Signal. 15, 1583–1606. 10.1089/ars.2011.3999 21473702 PMC3151424

[B35] Galiè N. Ghofrani A. H. (2013). New horizons in pulmonary arterial hypertension therapies. Eur. Respir. Rev. 22, 503–514. 10.1183/09059180.00006613 24293466 PMC9639192

[B36] Galiè N. Hoeper M. M. Humbert M. Torbicki A. Vachiery J. L. Barbera J. A.. (2009). Guidelines for the diagnosis and treatment of pulmonary hypertension. Eur. Heart J. 30, 2493–2537. 10.1093/eurheartj/ehp297 19713419

[B37] Galiè N. Humbert M. Vachiery J. L. Gibbs S. Lang I. Torbicki A.. (2016). 2015 ESC/ERS Guidelines for the diagnosis and treatment of pulmonary hypertension. Eur. Heart J. 37, 67–119. 10.1093/eurheartj/ehv317 26320113

[B38] Gentile C. L. Weir T. L. (2018). The gut microbiota at the intersection of diet and human health. Science 362, 776–780. 10.1126/science.aau5812 30442802 PMC13264711

[B39] Ghofrani H. A. D'Armini A. M. Grimminger F. Hoeper M. M. Jansa P. Kim N. H.. (2013). Riociguat for the treatment of chronic thromboembolic pulmonary hypertension. N. Engl. J. Med. 369, 319–329. 10.1056/NEJMoa1209657 23883377

[B40] Goveia J. Stapor P. Carmeliet P. (2014). Principles of targeting endothelial cell metabolism to treat angiogenesis and endothelial cell dysfunction in disease. EMBO Mol. Med. 6, 1–16. 10.15252/emmm.201404156 25063693 PMC4197858

[B41] Graham M. J. Lee R. G. Brandt T. A. Hurh E. Paz E. Mcevoy B. W.. (2017). Cardiovascular and metabolic effects of ANGPTL3 antisense oligonucleotides. N. Engl. J. Med. 377, 222–232. 10.1056/NEJMoa1701329 28538111

[B42] Griffin N. W. Ahern P. P. Cheng J. Heath A. C. Ilkayeva O. Newgard C. B.. (2017). Prior dietary practices and connections to a human gut microbial metacommunity alter responses to diet interventions. Cell Host Microbe 21, 123–125. 10.1016/j.chom.2016.12.006 28041931 PMC5234936

[B43] Grootaert M. O. da Costa Martins P. A. Bitsch N. Pintelon I. De Meyer G. R. Martinet W.. (2015). Defective autophagy in vascular smooth muscle cells accelerates senescence and promotes neointima formation and atherogenesis. Autophagy 11, 2014–2032. 10.1080/15548627.2015.1096485 26391655 PMC4824610

[B44] Hansson G. K. (2005). Inflammation, atherosclerosis, and coronary artery disease. N. Engl. J. Med. 352, 1685–1695. 10.1056/NEJMra043430 15843671

[B45] Harvey L. D. Chan S. Y. (2017). Emerging metabolic therapies in pulmonary arterial hypertension. J. Clin. Med. 6:43. 10.3390/jcm6040043 28375184 PMC5406775

[B46] Hernandez-Saavedra D. Swain K. Tuder R. Petersen S. V. Nozik-grayck E. (2017). Redox regulation of the superoxide dismutases SOD3 and SOD2 in the pulmonary circulation. Adv Exp Med Biol. 967, 57–70. 10.1007/978-3-319-63245-2 29047081

[B47] Hoefer I. E. Steffens S. Ala-Korpela M. Bäck M. Badimon L. Bochaton-Piallat M. L.. (2015). Novel methodologies for biomarker discovery in atherosclerosis. Eur. Heart J. 36, 2635–2642. 10.1093/eurheartj/ehv236 26049157

[B48] Hoeper M. M. (2015). Pharmacological therapy for patients with chronic thromboembolic pulmonary hypertension. Eur. Respir. Rev. 24, 272–282. 10.1183/16000617.00001015 26028639 PMC9487825

[B49] Hornberg J. J. Bruggeman F. J. Bakker B. M. Westerhoff H. V. (2007). Metabolic control analysis to identify optimal drug targets, in Systems Biological Approaches in Infectious Diseases. Progress in Drug Research, Vol 64, eds Boshoff H. I. Barry C. E (Basel: Birkhäuser Basel). 10.1007/978-3-7643-7567-6 17195475

[B50] Humbert M. (2010). Pulmonary arterial hypertension and chronic thromboembolic pulmonary hypertension: pathophysiology. Eur. Respir. Rev. 19, 59–63. 10.1183/09059180.00007309 20956167 PMC9491634

[B51] Humbert M. Lau E. M. Montani D. Jaïs X. Sitbon O. Simonneau G. (2014). Advances in therapeutic interventions for patients with pulmonary arterial hypertension. Circulation 130, 2189–2208. 10.1161/CIRCULATIONAHA.114.006974 25602947

[B52] Humbert M. Morrell N. W. Archer S. L. Stenmark K. R. MacLean M. R. Lang I. M.. (2004). Cellular and molecular pathobiology of pulmonary arterial hypertension. J. Am. Coll. Cardiol. 43, S13–S24. 10.1016/j.jacc.2004.02.029 15194174

[B53] Husted S. De Caterina R. Andreotti F. Arnesen H. Bachmann F. Huber K. (2014). Non-vitamin K antagonist oral anticoagulants (NOACs): no longer new or novel. Thromb. Haemost. 111, 781–782. 10.1160/TH14-03-0228 24658395

[B54] Hyder J. A. Allison M. A. Criqui M. H. Wright C. M. (2007). Association between systemic calcified atherosclerosis and bone density. Calcif. Tissue Int. 80, 301–306. 10.1007/s00223-007-9004-6 17505774

[B55] Ibanez B. James S. Agewall S. Antunes M. J. Bucciarelli-Ducci C. Bueno H.. (2018). 2017 ESC Guidelines for the management of acute myocardial infarction in patients presenting with ST-segment elevation. Eur. Heart J. 39, 119–177. 10.1093/eurheartj/ehx393 29198432

[B56] Jeffery T. K. Morrell N. W. (2002). Molecular and cellular basis of pulmonary vascular remodeling in pulmonary hypertension. Prog. Cardiovasc. Dis. 45, 173–202. 10.1053/pcad.2002.130041 12525995

[B57] Jenkins D. (2015). Pulmonary endarterectomy: the potentially curative treatment for patients with chronic thromboembolic pulmonary hypertension. Eur. Respir. Rev. 24, 263–271. 10.1183/16000617.00000815 26028638 PMC9487822

[B58] Kanhai D. A. Visseren F. L. Van Der Graaf Y. Schoneveld A. H. Catanzariti L. M. Timmers L.. (2013). Microvesicle protein levels are associated with increased risk for future vascular events and mortality in patients with clinically manifest vascular disease. Int. J. Cardiol. 168, 2358–2363. 10.1016/j.ijcard.2013.01.231 23484740

[B59] Kaptoge S. Di Angelantonio E. Pennells L. Wood A. M. White I. Gao P.. (2012). C-reactive protein, fibrinogen, and cardiovascular disease prediction. N. Engl. J. Med. 367, 1310–1320. 10.1056/NEJMoa1107477 23034020 PMC3714101

[B60] Kell D. B. Goodacre R. (2014). Metabolomics and systems pharmacology: why and how to model the human metabolic network for drug discovery. Drug Discov. Today 19, 171–182. 10.1016/j.drudis.2013.07.014 23892182 PMC3989035

[B61] Klarin D. Damrauer S. M. Cho K. Sun Y. V. Teslovich T. M. Honerlaw J.. (2018). Genetics of blood lipids among 300000 multi-ethnic participants of the Million Veteran Program. Nat. Genet. 50, 1514–1523. 10.1038/s41588-018-0222-9 30275531 PMC6521726

[B62] Lacroix R. Robert S. Poncelet P. Kasthuri R. S. Key N. S. Dignat-George F. (2010). Standardization of platelet-derived microparticle enumeration by flow cytometry with calibrated beads: results of the International Society on Thrombosis and Haemostasis SSC Collaborative workshop. J. Thromb. Haemost. 8, 2571–2574. 10.1111/j.1538-7836.2010.04047.x 20831623

[B63] Lang I. (2015). Chronic thromboembolic pulmonary hypertension: a distinct disease entity. Eur. Respir. Rev. 24, 246–252. 10.1183/16000617.00001115 26028636 PMC9487810

[B64] Lang I. M. Madani M. (2014). Update on chronic thromboembolic pulmonary hypertension. Circulation 130, 508–518. 10.1161/CIRCULATIONAHA.114.009309 25092279

[B65] Leopold J. A. Loscalzo J. (2005). Oxidative enzymopathies and vascular disease. Arterioscler. Thromb. Vasc. Biol. 25, 1332–1340. 10.1161/01.ATV.0000163846.51473.09 15790928

[B66] Lewis G. D. (2014). The emerging role of metabolomics in the development of biomarkers for pulmonary hypertension and other cardiovascular diseases (2013 Grover Conference Series). Pulm. Circ. 4, 417–423. 10.1086/677369 25621155 PMC4278601

[B67] Li H. Horke S. Förstermann U. (2014). Vascular oxidative stress, nitric oxide and atherosclerosis. Atherosclerosis 237, 208–219. 10.1016/j.atherosclerosis.2014.09.001 25244505

[B68] Liu W. Zhang Y. Yu C. M. Ji Q. W. Cai M. Zhao Y. X.. (2015). Current understanding of coronary artery calcification. J. Geriatr. Cardiol. 12, 668–675. 10.11909/j.issn.1671-5411.2015.06.012 26788045 PMC4712374

[B69] Lu Y. Zhang L. Liao X. Sangwung P. Prosdocimo D. A. Zhou G.. (2013). Kruppel-like factor 15 is critical for vascular infammation. J. Clin. Invest. 123, 4232–4241. 10.1172/JCI68552 23999430 PMC3785338

[B70] Makino A. Firth A. L. Yuan J. X. (2017). Endothelial and smooth muscle cell ion channels in pulmonary vasoconstriction and vascular remodeling. Compr. Physiol. 1, 1555–1602. 10.1002/cphy.c100023 23733654 PMC5524522

[B71] Masri F. A. Xu W. Comhair S. A. Asosingh K. Koo M. Vasanji A.. (2007). Hyperproliferative apoptosis-resistant endothelial cells in idiopathic pulmonary arterial hypertension. Am. J. Physiol. Lung Cell. Mol. Physiol. 293, 548–554. 10.1152/ajplung.00428.2006 17526595

[B72] Matsumoto S. Sakata Y. Suna S. Nakatani D. Usami M. Hara M.. (2013). Circulating p53-responsive MicroRNAs are predictive indicators of heart failure after acute myocardial infarction. Circ. Res. 113, 322–326. 10.1161/CIRCRESAHA.113.301209 23743335

[B73] McMurtry M. S. (2004). Dichloroacetate prevents and reverses pulmonary hypertension by inducing pulmonary artery smooth muscle cell apoptosis. Circ. Res. 95, 830–840. 10.1161/01.RES.0000145360.16770.9f 15375007

[B74] Michelakis E. D. Gurtu V. Webster L. Barnes G. Watson G. Howard L.. (2017). Inhibition of pyruvate dehydrogenase kinase improves pulmonary arterial hypertension in genetically susceptible patients. Sci. Transl. Med. 9:eaao4583. 10.1126/scitranslmed.aao4583 29070699

[B75] Moreno-Sánchez R. Rodríguez-Enríquez S. Marín-Hernández A. Saavedra E. (2007). Energy metabolism in tumor cells. FEBS J. 274, 1393–1418. 10.1111/j.1742-4658.2007.05686.x 17302740

[B76] Moreno-Viedma V. Amor M. Sarabi A. Bilban M. Staffler G. Zeyda M.. (2016). Common dysregulated pathways in obese adipose tissue and atherosclerosis. Cardiovasc. Diabetol. 15, 120. 10.1186/s12933-016-0441-2 27561966 PMC5000404

[B77] Münzel T. Camici G. G. Maack C. Bonetti N. R. Fuster V. Kovacic J. C. (2017). Impact of oxidative stress on the heart and vasculature. J. Am. Coll. Cardiol. 70, 212–229. 10.1016/j.jacc.2017.05.035 28683969 PMC5663297

[B78] Münzel T. Gori T. Bruno R. M. Taddei S. (2010). Translational medicine Is oxidative stress a therapeutic target in cardiovascular disease ? Eur. Heart J. 31, 2741–2749. 10.1093/eurheartj/ehq396 20974801

[B79] Nilsson A. Nielsen J. (2016). Genome scale metabolic modeling of cancer. Metab. Eng. 43, 103–112. 10.1016/j.ymben.2016.10.022 27825806

[B80] Nogueira-Ferreira R. Ferreira R. Henriques-Coelho T. (2014). Cellular interplay in pulmonary arterial hypertension: implications for new therapies. Biochim. Biophys. Acta Mol. Cell Res. 1843, 885–893. 10.1016/j.bbamcr.2014.01.030 24491811

[B81] Pangare M. Makino A. (2012). Mitochondrial function in vascular endothelial cell in diabetes. J. Smooth Muscle Res. 48, 1–26. 10.1540/jsmr.48.1 22504486 PMC3655204

[B82] Parra-bonilla G. Alvarez D. F. Al-Mehdi A. B. Alexeyev M. Stevens T. (2010). Critical role for lactate dehydrogenase A in aerobic glycolysis that sustains pulmonary microvascular endothelial cell proliferation. Am. J. Physiol. Lung Cell. Mol. Physiol. 299, 513–522. 10.1152/ajplung.00274.2009 20675437 PMC2957419

[B83] Parsa C. J. Daneshmand M. A. Gaca J. C. Rankin J. S. (2011). Arterial bypass grafting of the coronary circulation. HSR Proc. Intensive Care Cardiovasc. Anesth. 3, 227–234. 23439991 PMC3563437

[B84] Patra K. C. Hay N. (2014). The pentose phosphate pathway and cancer. Trends Biochem. Sci. 39, 347–354. 10.1016/j.tibs.2014.06.005 25037503 PMC4329227

[B85] Piazza G. Goldhaber S. Z. (2011). Chronic thromboembolic pulmonary hypertension. N. Engl. J. Med. 364, 351–360. 10.1056/NEJMra0910203 21268727

[B86] Pober J. S. Min W. Bradley J. R. (2009). Mechanisms of endothelial dysfunction, injury, and death. Annu. Rev. Pathol. Mech. Dis. 4, 71–95. 10.1146/annurev.pathol.4.110807.092155 18754744

[B87] Quarck R. Wynants M. Ronisz A. Sepulveda M. R. Wuytack F. Van Raemdonck D.. (2012). Characterization of proximal pulmonary arterial cells from chronic thromboembolic pulmonary hypertension patients. Respir. Res. 13:27. 10.1186/1465-9921-13-27 22452949 PMC3352254

[B88] Rizzuto R. De Stefani D. Raffaello A. Mammucari C. (2012). Mitochondria as sensors and regulators of calcium signalling. Nat. Rev. Mol. Cell Biol. 13, 566–578. 10.1038/nrm3412 22850819

[B89] Rohatgi A. Khera A. Berry J. D. Givens E. G. Ayers C. R. (2014). HDL Cholesterol efflux capacity and incident cardiovascular events. N. Engl. J. Med. 371, 2383–2393. 10.1037/a0018493 25404125 PMC4308988

[B90] Ryan J. J. Archer S. L. (2015). Emerging concepts in the molecular basis of pulmonary arterial hypertension (PAH): Part I: metabolic plasticity and mitochondrial dynamics in the pulmonary circulation and right ventricle in PAH. Circulation 131, 1691–1702. 10.1161/CIRCULATIONAHA.114.006979 25964279 PMC4429908

[B91] Ryu J. Y. Kim H. U. Lee S. Y. (2015). Reconstruction of genome-scale human metabolic models using omics data. Integr. Biol. 7, 859–868. 10.1039/C5IB00002E 25730289

[B92] Sandoo A. van Zanten J. J. Metsios G. S. Carroll D. Kitas G. D. (2010). The endothelium and its role in regulating vascular tone. Open Cardiovasc. Med. J. 4, 302–312. 10.2174/1874192401004010302 21339899 PMC3040999

[B93] Santos M. J. Pedro L. M. Canhão H. Fernandes E Fernandes J. Canas da Silva J. Fonseca J. E.. (2011). Hemorheological parameters are related to subclinical atherosclerosis in systemic lupus erythematosus and rheumatoid arthritis patients. Atherosclerosis 219, 821–826. 10.1016/j.atherosclerosis.2011.08.026 21906736

[B94] Schoors S. De Bock K. Cantelmo A. R. Georgiadou M. Ghesquière B. Cauwenberghs S.. (2014). Partial and transient reduction of glycolysis by PFKFB3 blockade reduces pathological angiogenesis. Cell Metab. 19, 37–48. 10.1016/j.cmet.2013.11.008 24332967

[B95] Schrijvers D. M. De Meyer G. R. Kockx M. M. Herman A. G. Martinet W. (2005). Phagocytosis of apoptotic cells by macrophages is impaired in atherosclerosis. Arterioscler. Thromb. Vasc. Biol. 25, 1256–1261. 10.1161/01.ATV.0000166517.18801.a7 15831805

[B96] Semenza G. L. (2008). Tumor metabolism : cancer cells give and take lactate. J. Clin. Invest. 118, 3835–3837. 10.1128/IAI.01022-08.16 19033652 PMC2582934

[B97] Serino M. Blasco-baque V. Nicolas S. (2014). Far from the eyes, close to the heart : dysbiosis of gut microbiota and cardiovascular consequences. Curr. Cardiol. Rep. 16, 540. 10.1007/s11886-014-0540-1 25303894 PMC4194023

[B98] Shah S. H. Bain J. R. Muehlbauer M. J. Stevens R. D. Crosslin D. R. Haynes C.. (2010). Association of a peripheral blood metabolic profile with coronary artery disease and risk of subsequent cardiovascular events. Circ. Cardiovasc. Genet. 3, 207–214. 10.1161/CIRCGENETICS.109.852814 20173117

[B99] Sinha S. Iyer D. Granata A. (2014). Cellular and Molecular Life Sciences Embryonic origins of human vascular smooth muscle cells: implications for *in vitro* modeling and clinical application. Cell. Mol. Life Sci. 71, 2271–2288. 10.1007/s00018-013-1554-3 24442477 PMC4031394

[B100] Sitia S. Tomasoni L. Atzeni F. Ambrosio G. Cordiano C. Catapano A.. (2010). From endothelial dysfunction to atherosclerosis. Autoimmun. Rev. 9, 830–834. 10.1016/j.autrev.2010.07.016 20678595

[B101] Stacpoole P. W. Nagaraja N. V. Hutson A. D. (2003). Efficacy of dichloroacetate as a lactate-lowering drug. J. Clin. Pharmacol. 43, 683–691. 10.1177/0091270003254637 12856382

[B102] Steffel J. Verhamme P. Potpara T. S. Albaladejo P. Antz M. Desteghe L. (2018). The 2018 European Heart Rhythm Association Practical Guide on the use of non-vitamin K antagonist oral anticoagulants in patients with atrial fibrillation: executive summary. Europace. 20, 1231–1242. 10.1093/europace/euy054 29562331

[B103] Stegemann C. Pechlaner R. Willeit P. Langley S. R. (2014). Lipidomics profiling and risk of cardiovascular disease in the prospective population-based bruneck study christin. Circulation 129, 1821–1831. 10.1161/CIRCULATIONAHA.113.002500 24622385

[B104] Stenmark K. R. Davie N. Frid M. Gerasimovskaya E. Das M. (2006). Role of the adventitia in pulmonary vascular remodeling. Physiology 21, 134–145. 10.1152/physiol.00053.2005 16565479

[B105] Suganuma K. Miwa H. Imai N. Shikami M. Gotou M. Goto M.. (2010). Energy metabolism of leukemia cells: glycolysis versus oxidative phosphorylation. Leuk. Lymphoma 51, 2112–2119. 10.3109/10428194.2010.512966 20860495

[B106] Tang W. H. Kitai T. Hazen S. L. (2017). Gut microbiota in cardiovascular health and disease. Circ. Res. 120, 1183–1196. 10.1161/CIRCRESAHA.117.309715 28360349 PMC5390330

[B107] Townsend N. Nichols M. Scarborough P. Rayner M. (2015). Cardiovascular disease in Europe - Epidemiological update 2015. Eur. Heart J. 36, 2696–2705. 10.1093/eurheartj/ehv428 26306399

[B108] Townsend N. Wilson L. Bhatnagar P. Wickramasinghe K. Rayner M. Nichols M. (2016). Cardiovascular disease in Europe: epidemiological update 2016. Eur. Heart J. 37, 3232–3245. 10.1093/eurheartj/ehw334 27523477 PMC13480964

[B109] Vander Heiden M. G. Cantley L. C. Thompson C. B. (2009). Understanding the Warburg effect: the metabolic Requiremetns of cell proliferation. Science 324, 1029–1033. 10.1126/science.1160809 19460998 PMC2849637

[B110] Vega-Naredo I. Loureiro R. Mesquita K. A. Barbosa I. A. Tavares L. C. Branco A. F.. (2014). Mitochondrial metabolism directs stemness and differentiation in P19 embryonal carcinoma stem cells. Cell Death Differ. 21, 1560–1574. 10.1038/cdd.2014.66 24832466 PMC4158682

[B111] Ward-Caviness C. K. Xu T. Aspelund T. Thorand B. Montrone C. Meisinger C.. (2017). Improvement of myocardial infarction risk prediction via inflammation-associated metabolite biomarkers. Heart 103, 1278–1285. 10.1136/heartjnl-2016-310789 28255100 PMC5871235

[B112] Willer C. J. Schmidt E. M. Sengupta S. Peloso G. M. Gustafsson S. (2013). Discovery and refinement of Loci Associated with Lipid Levels. Nat. Genet. 45, 1274–1283. 10.1038/ng.2797 24097068 PMC3838666

[B113] Xia N. Daiber A. Förstermann U. Li H. (2017). Antioxidant effects of resveratrol in the cardiovascular system. Br. J. Pharmacol. 174, 1633–1646. 10.1111/bph.13492 27058985 PMC5446570

[B114] Xu W. Erzurum S. C. (2011). Endothelial cell energy metabolism, proliferation, and apoptosis in pulmonary hypertension. Compr. Physiol. 1, 357–372. 10.1002/cphy.c090005 23737177 PMC6467053

[B115] Xu W. Koeck T. Lara A. R. Neumann D. DiFilippo F. P. Koo M.. (2007). Alterations of cellular bioenergetics in pulmonary artery endothelial cells. Proc. Natl. Acad. Sci. U.S.A. 104, 1342–1347. 10.1073/pnas.0605080104 17227868 PMC1783136

[B116] Xu Y. Wang Y. Yan S. Yang Q. Zhou Y. Zeng X.. (2017). Regulation of endothelial intracellular adenosine via adenosine kinase epigenetically modulates vascular inflammation. Nat. Commun. 8:943. 10.1038/s41467-017-00986-7 29038540 PMC5643397

[B117] Yilmaz L. S. Walhout A. J. (2017). Metabolic network modeling with model organisms. Curr. Opin. Chem. Biol. 36, 32–39. 10.1016/j.cbpa.2016.12.025 28088694 PMC5458607

[B118] Young J. D. (2014). INCA: a computational platform for isotopically non-stationary metabolic flux analysis. Bioinformatics 30, 1333–1335. 10.1093/bioinformatics/btu015 24413674 PMC3998137

[B119] Zardi E. M. Afeltra A. (2010). Endothelial dysfunction and vascular stiffness in systemic lupus erythematosus: are they early markers of subclinical atherosclerosis? Autoimmun. Rev. 9, 684–686. 10.1016/j.autrev.2010.05.018 20553974

[B120] Zhang C. Hua Q. (2016). Applications of genome-scale metabolic models in biotechnology and systems medicine. Front. Physiol. 6:413. 10.3389/fphys.2015.00413 26779040 PMC4703781

[B121] Zhao Y. Peng J. Lu C. Hsin M. Mura M. Wu L.. (2014). Metabolomic heterogeneity of pulmonary arterial hypertension. PLoS ONE 9:e88727. 10.1371/journal.pone.0088727 24533144 PMC3923046

